# Hypoglycaemia Risk Prediction Models for Type 2 Diabetes: A Systematic Review and Meta‐Analysis

**DOI:** 10.1002/edm2.70227

**Published:** 2026-04-29

**Authors:** Yiwen Wei, Yu Liu, Jingwen Bo, Xiaoyan Bai

**Affiliations:** ^1^ School of Nursing Beijing University of Chinese Medicine Beijing China

**Keywords:** hypoglycaemia, risk prediction model, systematic review, Type 2 diabetes mellitus

## Abstract

**Background:**

The growing number of hypoglycaemia risk prediction models for Type 2 diabetes mellitus (T2DM) underscores the need for systematic evaluation of their risk of bias and applicability. This study summarises and critically assesses their characteristics and predictive performance using established guidelines for prediction model development.

**Methods:**

The review protocol was registered on PROSPERO (CRD420251031980). We searched nine main English and Chinese databases from inception to May 2025. The CHARMS checklist and PROBAST tool were used to assess the risk of bias and applicability. A meta‐analysis of AUC values from models was conducted using MedCalc software.

**Results:**

We included 25 studies (45 models), with reported AUCs ranging from 0.630 to 0.996. The pooled AUC value of 16 models was 0.815 (95% CI 0.765–0.861), indicating excellent discrimination. 24 (96%) studies were overall at high risk of bias and 22 (88%) studies had low‐risk applicability, primarily due to small sample size, improper handling of missing data, failure to report calibration, screening of predictors by univariate analysis and lack of external validation.

**Conclusions:**

Current hypoglycaemia prediction models for T2DM show substantial methodological limitations and high bias risk. While machine learning models have advanced rapidly in recent years, their methodology remains opaque and validation is limited. Future research should focus on optimising existing models, enhancing methodological rigour and conducting external validation.

## Graphical abstract

This systematic review and meta‐analysis evaluated existing hypoglycaemia risk prediction models for Type 2 diabetes, finding excellent pooled discrimination (AUC = 0.815) but widespread high methodological bias, highlighting the need for rigorous model optimisation and external validation.

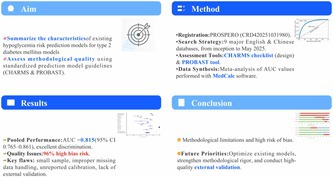

## 1 Introduction

Hypoglycaemia is one of the most common acute complications of Type 2 diabetes mellitus (T2DM) and a key factor in glycaemic management. Strict blood glucose restriction, inappropriate use of hypoglycaemic agents, dietary control and other factors make patients with T2DM susceptible to hypoglycaemic reactions [1]. The incidence of hypoglycaemia in patients with T2DM is relatively high, ranging from 22.5% to 31.6% [2, 3]. In 2013, a cross‐sectional survey of 6713 T2DM patients in 27 provinces and municipalities directly under the central government in China showed that the incidence of hypoglycaemia was 20.99% (1409/6713), including 1296 cases of mild hypoglycaemia and 14 cases of severe hypoglycaemia [4]. In 2016, a multicentre cross‐sectional survey of 3167 hospitalised adults with T2DM in Italy found that the incidence of hypoglycaemia during hospitalisation was 28% in 887 patients, and the occurrence of hypoglycaemia prolonged the average length of hospital stay and increased in‐hospital mortality [5]. In 2020, a cross‐sectional survey of 618 adult T2DM patients in the Spanish community showed a hypoglycaemia incidence of 30.4% (188/618), with 19.1% loss of consciousness due to hypoglycaemic episodes [6].

Hypoglycaemia has a broad range of negative effects on the health of patients with diabetes, such as causing functional damage to the heart, brain, blood vessels, kidneys and other organs of patients, leading to anxiety and severe hypoglycaemia and even endangering the lives of patients [7]. There are many factors that affect the occurrence of hypoglycaemia in the patients with T2DM, roughly divided into three aspects: demographic factors, disease factors and lifestyle factors, such as age, insulin use, glycated haemoglobin levels and history of hypoglycaemia [8, 9, 10]. Early identification of the influencing factors of hypoglycaemia is of great significance for effectively preventing hypoglycaemia in patients with T2DM. Therefore, diabetes researchers constructed hypoglycaemic risk prediction models to predict the risk of hypoglycaemia occurrence and intervene in advance.

A predictive model is a mathematical equation involving multiple variables that estimate the probability of individuals in a specific health state developing specific health outcomes. These models assist healthcare professionals in better preventing and identifying the outcome events and further optimising diagnostic and treatment strategies [11]. In 2022, a systematic review published in China on hypoglycaemia risk prediction models for diabetic patients included 14 studies and 16 hypoglycaemia risk prediction models. The results indicated that these models generally demonstrated satisfactory discrimination, calibration performance and applicability, yet they exhibited significant methodological flaws and a high risk of bias. Although this systematic review broadly analysed hypoglycaemia risk prediction models across diabetes populations, it lacked a dedicated systematic evaluation specifically focused on hypoglycaemia risk prediction models for patients with T2DM [12]. In 2023, another Chinese systematic review of hypoglycaemia risk prediction models for patients with T2DM included 9 studies and 12 models, and the results showed that the model‐building methods in these studies were predominantly based on logistic regression [13]. Since 2023, research activity in this field has increased markedly, closely paralleling the growing adoption of machine learning algorithms. Therefore, it is necessary to incorporate these latest studies to provide an updated evidence synthesis.

In this study, we systematically searched the existing research on the risk prediction models of hypoglycaemia in patients with T2DM. Guided by established prediction model development standards [14], we standardised the assessment of their risk of bias and applicability and conducted a comprehensive summary and critical evaluation of their characteristics and predictive performance.

## 2 Methods

### 2.1 Retrieval Strategy

The review was registered with the International Prospective Register of Systematic Reviews (PROSPERO)(CRD420251031980). Two of our team systematically searched PubMed, the Cochrane Library, CINAHL, Web of Science, ProQuest, SinoMed, CNKI, Wanfang Data and VIP Database from inception to May 2025. The following keywords were used for the basic search: ‘Diabetes Mellitus’, ‘Type 2’, ‘Type 2 diabetes’, ‘diabetes, Type 2’, ‘T2DM’, ‘2‐dm’, ‘hypoglycaemia’, ‘glycopenia’, ‘risk prediction’, ‘risk evaluation’, ‘risk assessment’, ‘prediction model’, ‘predict’, ‘tool’, ‘Scale’, ‘score’. The complete registration and search strategies are presented in Data S1.

### 2.2 Eligibility Criteria

We followed the PICOTS system to formulate our review question as follows [15]: (1) Which models developed and/or validated are currently available to predict the risk of hypoglycaemia in patients with T2DM? (2) How do these models rate for performance and applicability? The PICOTS statement was as follows:

*P (Population)*: Patients with diagnosed T2DM.

*I (Intervention)*: All available hypoglycaemia risk prediction models.

*C (Comparator)*: Not applicable.

*O (Outcome)*: Occurrence of hypoglycaemia.

*T (Timing)*: Time from baseline survey to the onset of hypoglycaemia in the patient, or the occurrence of hypoglycaemia during a specified period.

*S (Setting)*: The contexts in which risk prediction models are used, including inpatient, outpatient, acute care, community‐based and home‐based settings.

*Inclusion criteria*: (1) Research Objective: patients with T2DM (including T2DM complications). (2) Study design: cohort studies, cross‐sectional studies and randomised controlled studies. (3) Outcome: the occurrence of hypoglycaemia, including symptomatic hypoglycaemia, asymptomatic hypoglycaemia and hypoglycaemia‐related events. (4) Study content: construction or validation of hypoglycaemia risk prediction models for patients with T2DM, describing in detail the process of model construction, performance and validation.

*Exclusion criteria*: (1) Type 1 diabetes mellitus, gestational diabetes mellitus, or other special diabetes mellitus. (2) Did not report the occurrence of hypoglycaemia. (3) Reviews, commentaries, conference abstracts and news reports. (4) Did not describe the model construction process and method. (5) The full text was not available.

### 2.3 Study Selection and Data Extraction

Software Endnote was used to import the retrieved studies and remove duplicates. Two reviewers independently applied review questions and eligibility criteria to screen studies. If consensus was not achieved, a third reviewer was invited until unity of opinion was reached. Once the included studies were identified, reviewers used a standardised data extraction form based on the Checklist for Critical appraisal and data extraction for systematic Reviews of prediction modelling studies (CHARMS) [16], including the first author, year, country, study design, participants, sample size (calculation method), predicted outcome, modelling method, variable selection, candidate and included predictors, predictive performance measures, model validation, handling of missing data, etc. The data extraction process was conducted by two reviewers. In cases of disagreement during data extraction, a third reviewer was invited to help reach a consensus through discussion.

### 2.4 Risk of Bias and Applicability Assessment

The risk of bias and applicability was independently assessed by two reviewers using the PROBAST (Prediction Model Risk of Bias Assessment Tool) [17]. The tool contains 20 signalling questions in four domains: participants, predictors, outcome and analysis. Each signalling question can be answered as ‘yes, probably yes, no, probably not, no information’. The bias risk of each domain and the overall prediction model were determined according to the answer to the signalling questions. The applicability assessment contains three domains: participants, predictors and outcome. The applicability of the prediction model in each domain and as a whole was assessed based on the descriptions, which can be graded as low, high, or unclear.

### 2.5 Data Synthesis and Statistical Analysis

A meta‐analysis of the area under the curve (AUC) values from models was conducted using MedCalc software (version20.0). Heterogeneity was tested using the *I* ^2^ index and Cochrane *Q* test [18]. Fixed or random effects models were used based on the heterogeneity of the analysis results, and Egger's test was used to identify publication bias, with *p* > 0.05 indicating a low likelihood of publication bias [19].

## 3 Results

**FIGURE 1:**
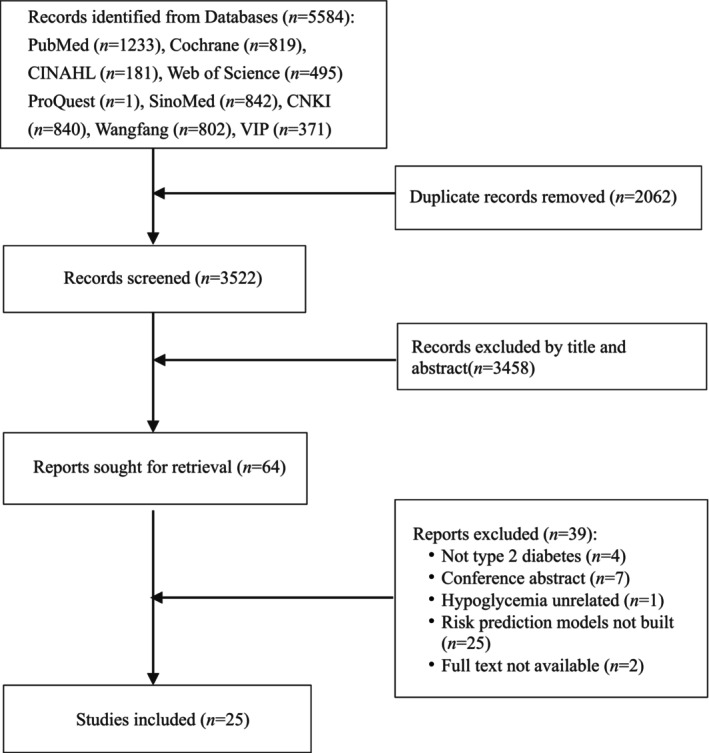
Flowchart of literature search and selection.

As shown in Figure 1, a total of 5584 relevant papers were searched, 3522 papers were initially included after removing duplicate records, and 25 studies were finally included after progressive screening.

**FIGURE 2:**
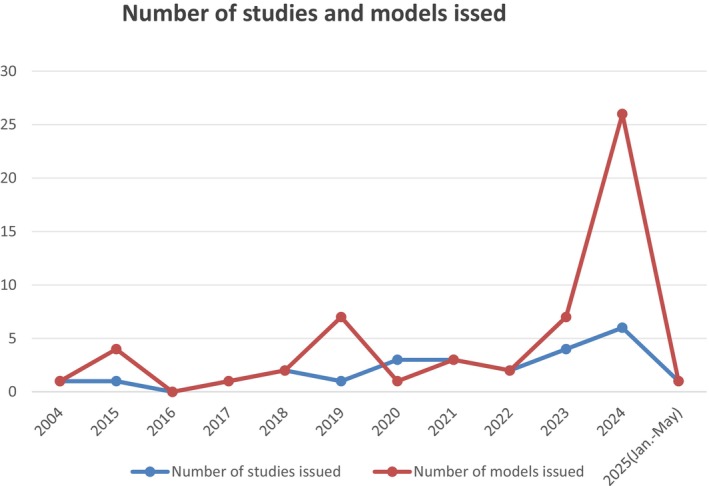
Number of studies and models issued.

Table 1 lists the detailed features of the 25 included studies. They were published between 2004 and 2025, and the number of articles has gradually increased over the past 20 years, with 11 published (44%) in the past two and a half years, reaching its peak in 2024 (Figure 2). Of the included studies, 12 were from China, 8 from Europe and the United States, and 1 each from Korea, Qatar, Australia, India and Denmark. 20 studies were cohort studies (14 were retrospective and 6 were prospective), 4 were cross‐sectional studies and 1 was RCT. Nine studies (36%) reported the prediction horizon of the model (3 months to 5 years). The included studies all reported the outcome (occurrence of hypoglycaemia) and the diagnostic criteria of outcome.

**TABLE 1:** Characteristics of included studies (*n* = 25).

Study	Country	Study design	Published	Model type	Participants	Outcome measurements	Prediction horizon	Diagnosis criteria
Murata et al. [20]	USA	Cross‐sectional	2004	Development and validation	T2DM patients: diagnosed as T2DM ≥ 2 months	Hypoglycaemia	1 year	Hypoglycaemia: glucose level of 60 mg/dL or ≤ 3.33 mmol/L
Sudharsan et al. [21]	USA	Cross‐sectional	2015	Development and validation	T2DM patients	Hypoglycaemia	NR	Hypoglycaemia: SMBG < 70 mg/dL
Karter et al. [22]	USA	Prospective cohort	2017	Development and validation	T2DM patients seeking emergency treatment or hospitalisation	Hypoglycaemia	1 year	Hypoglycaemia: hypoglycaemia according to ICD‐9 criteria
Chow et al. [23]	USA	Prospective cohort	2018	Development and validation	T2DM patients: receiving treatment ≥ 3 months	Severe hypoglycaemia	5 years	Severe hypoglycaemia: hypoglycaemia requiring medical assistance, BG < 2.8 mmol/L or glucose administration
Kyungdo Han et al. [24]	Korea	Retrospective cohort	2018	Development and validation	T2DM patients	At least one episode of hypoglycaemia in the previous 3 years	1 year	Severe hypoglycaemia: hypoglycaemia according to International Classification of Diseases, Tenth Revision (ICD‐10) codes
Misra‐Hebert et al. [25]	USA	Retrospective cohort	2019	Development and validation	T2DM patients with prior non‐severe hypoglycaemia	Severe hypoglycaemia	3 months	Severe hypoglycaemia: hypoglycaemia according to ICD 9, 10
Heller et al. [26]	UK	RCT	2020	Development and validation	T2DM patients with high cardiovascular disease risk	Severe hypoglycaemia	2 years	Severe hypoglycaemia: hypoglycaemia requiring medical assistance, or other corrective action
Hu et al. [27]	China	Retrospective cohort	2020	Development and validation	Hospitalised adult T2DM patients receiving intensive insulin therapy	Occurrence during hospitalisation hypoglycaemia	NR	BG ≤ 3.9 mmol/L during hospitalisation
T. Elhadd et al. [28]	Doha	Prospective cohort	2020	Development and validation	18 ~ 79 year‐old T2DM patients	Hypoglycaemia from fasting during Ramadan	NR	Hypoglycaemia during fasting
Crutzen et al. [29]	Netherlands	Retrospective cohort	2021	Development and validation	T2DM patients: diagnosed as T2DM > 6 months, used hypoglycemic drugs > 1 year	Hypoglycaemia	NR	Hypoglycaemia: International Classification for Primary Care (T87) or BG measurement < 3.9 mmol/L
Kyi et al. [30]	Australia	Retrospective cohort	2021	Development and validation	Adults with T2DM	Persistent onset of hypoglycaemia	NR	Persistent hypoglycaemia: occurrence ≥ 2 days, capillary BG < 4 mmol/L
Zuo et al. [31]	China	Retrospective cohort	2021	Development and validation	Hospitalised T2DM patients	Hypoglycaemia	NR	Bedside fingertip glucose monitoring method; hypoglycaemia: BG ≤ 3.9 mmol/L with palpitations, anxiety, sweating, etc., coma, convulsions, and cognitive deficits; or daily glucose monitoring ≤ 3.9 mmol/L without symptoms
Han et al. [32]	China	Retrospective cohort	2022	Development and validation	T2DM patients hospitalised for elective surgery	Hypoglycaemia in the perioperative period of hospitalisation	NR	Grade 1 hypoglycaemia: glucose concentration 54 (3.0 mmol/L) ~ 70 mg/dL (3.9 mmol/L) Grade 2 hypoglycaemia: BG < 54 mg/dL (3.0 mmol/L)
Yang et al. [33]	China	Retrospective cohort study	2022	Development and validation	T2DM patients	Hypoglycaemia	NR	Hypoglycaemia: BG < 3.9 mmol/L or 70 mg/dL
Liu et al. [34]	China	Retrospective cohort	2023	Development	T2DM patients	Postoperative hypoglycaemia	NR	Postoperative hypoglycaemia: BG < 3.9 mmol/L
Zhang et al. [35]	China	Prospective study	2023	Development and validation	Elderly patients with T2DM	Hypoglycaemia within a week	NR	Hypoglycaemia: Glycaemia < 3.9 mmol/L in elderly diabetic patients on medication
Zheng [36]	China	Retrospective cohort	2023	Development and validation	Hospitalised T2DM patients	Hypoglycaemia after 24 h of hospitalisation	NR	Hypoglycaemia: BG < 3.9 mmol/L
Xu et al. [37]	China	Prospective cohort	2023	Development	Hospitalised T2DM patients	Hypoglycaemia within 3 months, 6 months	3 or 6 months	Pseudo‐hypoglycaemia (with typical symptoms of hypoglycaemia, BG > 3.9 mmol/L, but approaching that level), symptomatic hypoglycaemia (with symptoms of hypoglycaemia, BG ≤ 3.9 mmol/L), A symptomatic hypoglycaemia (without symptoms of hypoglycaemia, BG ≤ 3.9 mmol/L), severe hypoglycaemia (without a clear glycaemic threshold, with severe cognitive deficits, needing assistance in correcting the condition)
Shao et al. [38]	China	Retrospective study	2024	Development and validation	T2DM patients	Hypoglycaemia	NR	Mild hypoglycaemia: BG = 54 ~ 70 mg/dL, severe hypoglycaemia: BG < 54
Chen et al. [39]	China	Retrospective cohort	2024	Development and validation	T2DM patients hospitalised for ≥ 24 h of continuous glucose monitoring	Hypoglycaemia occurs between 0:00 a.m. and 6:00 a.m.	NR	Nocturnal hypoglycaemia: BG ≤ 3.9 mmol/L between 0:00 am–6:00 am
Guo et al. [40]	China	Prospective study	2024	Development and validation	Outpatient and inpatient elderly with T2DM	Hypoglycaemia within 6 months	6 months	BG ≤ 3.9 mmol/L measured at any time or conscious symptoms of hypoglycaemia
Agraz et al. [41]	USA	Retrospective cohort	2024	Development and validation	T2DM patients	Severe hypoglycaemia	1 year	Severe hypoglycaemia: BG of 54 mg/dl or less, determined by whether the patient experiences loss of consciousness or requires medical assistance
Gaikwad et al. [42]	India	Cross‐sectional	2024	Development and validation	Hospitalised T2DM patients	Hypoglycaemia	NR	Hypoglycaemia: BG < 70 mg/dL or 3.9 mmol/L
Hou et al. [10]	China	Cross‐sectional	2024	Development and validation	Hospitalised elderly with T2DM	Hypoglycaemia	NR	Hypoglycaemia: BG ≤ 3.9 mmol/L
Thomsen et al. [43]	DK	Retrospective cohort	2025	Development and validation	Insulin‐treated patients with T2DM	Hypoglycaemia	NR	Hypoglycaemia: BG < 70 mg/dL or 3.9 mmol/L

Abbreviations: BG, blood glucose; NR, not reported.

Twenty‐five studies with 45 models were included, all of which were model development (validation) studies. Three studies [21, 38, 39] reported 3 models each, 1 study [36] reported 4 models, 1 study [28] reported 5 models and 1 study [42] reported 7 models and the other studies reported 1 model. The sample size ranged from 180 to 1,173,820, and 3 studies [10, 35, 39] reported sample size calculations. Over half of the prediction models were developed using machine learning techniques (*n* = 27, 60%), and the first article appeared in 2015, followed by a rapid increase in the number of articles.

Regarding the handling of missing data: Out of 45 models reported in 25 studies, 17 models (37.8%) reported methods for handling missing data, with 5 models using multiple imputation, 4 models using mean/median and Mode replacement [36], 2 models using random forest regression [33, 39] (i.e., leveraging the random forest regression algorithm to allocate samples with missing values to different branches as reasonably as possible to reach an optimal split point with negligible missing values), 1 model using the last observation or mean replacement [41], 1 model using LGBM model auto‐learning algorithm [39] and the remaining 4 models using direct exclusion. The details of model establishment are shown in Table 2.

**TABLE 2:** Construction of included studies (*n* = 25).

Study	Sample size	Sample size calculation methodology	Modelling method (number of models)	Candidate prediction number of factors	Amount of missing data and how it was handled
Murata et al. [20]	195	NR	LR (1)	2	NR
Sudharsan et al. [21]	10,814	NR	RF, SVM, KNN, Bayesian (4)	NR	NR
Karter et al. [22]	165,148	NR	Classification tree (1)	156	NR
Chow et al. [23]	5135	NR	Cox (1)	41	Direct exclusion
Kyungdo Han et al. [24]	1,173,820	NR	Cox (1)	14	Direct exclusion
Misra‐Hebert et al. [25]	1876	NR	LR (1)	14	Multiple imputation
Heller et al. [26]	1191	NR	Cox (1)	NR	NR
Hu et al. [27]	257	NR	LR (1)	18	NR
T. Elhadd et al. [28]	19,540	NR	Linear regression RF, XGboost, SVM deep learning (5)	55	NR
Crutzen et al. [29]	13,876	NR	LR (1)	43	Multiple imputation
Kyi et al. [30]	594	NR	LR (1)	13	NR
Zuo et al. [31]	1149	NR	LR (1)	15	NR
Han et al. [32]	1410	NR	LR (1)	10	NR
Yang et al. [33]	29,843	NR	XGboost (1)	176	RF
Liu et al. [34]	440	NR	LR (1)	26	NR
Zhang et al. [35]	300	EPV principle: 5 times the number of variables in the questionnaire.	RF (1)	35	Direct exclusion
Zheng [36]	612	NR	LR, XGboost, DT, RF (4)	19	Data with more than 25% missing values were excluded and replaced with mean or median for numeric variables and with plurality for categorical variables
Xu et al. [37]	1303	NR	LR (1)	Model 3 month: 18 Model 6 month: 18	Multiple imputation
Shao et al. [38]	192	NR	LSTM, SVM, RF (3)	NR	NR
Chen et al. [39]	440	Pmsampsize package to calculate sample size	LR, RF,OGEA (3)	28	LR Model: Multiple imputation RF Model: Optimal Segmentation Ignoring Missing Values LGBM model: automatically learns the data distribution and assigns default directions to missing values
Guo et al. [40]	277	NR	LR (1)	20	NR
Agraz et al. [41]	10,244	NR	Co‐training (1)	12	Time‐series observations are carried forward using the last observation, and non‐time‐series observations are interpolated using the median
Gaikwad et al. [42]	290	NR	GE, NN, AdaBoost, RF, DT, SVM, SGD (7)	NR	NR
Hou et al. [10]	585	Sample size calculation formula for cross‐sectional studies	LR (1)	24	Multiple imputation 20 times
Thomsen et al. [43]	180	NR	CNN (1)	NR	Direct exclusion

Abbreviations: Ada Boost, adaptive boosting; co‐training, co‐training machine learning models with multiple views; CNN, convolutional neural network; DT, decision tree; GE, gradient enhancement; KNN, K‐nearest neighbours; LSTM, long and short‐term memory networks; LR, logistic regression; NN, neural networks; NR, not reported; OGEA, optical gradient enhancer algorithm; RF, random forest; SGD, stochastic gradient descent; SVM, support vector machine; XGboost, eXtreme gradient boosting.

Regarding the model predictors: The number of predictors included in 45 models ranged from 2 to 37. The top five predictive factors were insulin use (*n* = 16), sulphonamide use (*n* = 12), age (*n* = 10), glycosylated haemoglobin (*n* = 10) and history of hypoglycaemia (*n* = 9). All studies reported results on model performance: 22 studies reported AUC ranging from 0.63 to 0.996, 1 study [21] using specificity and sensitivity, 1 study [28] using *R* ^2^ and mean absolute error and 1 study [41] using positive predictive value, accuracy and specificity.

Regarding model calibration: 8 studies reported using the Hosmer–Lemeshow test, 5 studies reported using calibration charts, 1 study reported using Brier Score and 11 studies did not report. Only 11 models (24.4%) were externally validated, 43 models (95.6%) were internally validated and 2 models (4.4%) were not validated. Of the internal validation models, the majority of these were cross‐validated using the Bootstrap method. Of the externally validated models, only 6 models reported the method and process of external validation. The predictors and performance of the models are assessed in Table 3.

**TABLE 3:** Predictors and performance of the models (*n* = 25).

Study	Variable selection	Risk factors included in models (*n*)	Model performance (differentiation)	Model performance (calibration)	Internal validation	External validation	Model presentation
Murata et al. [20]	Multifactorial analysis	Mean glucose, standard deviation (2)	0.746	NR	Risk stratification sampling	NR	NR
Sudharsan et al. [21]	Peak model accuracy	NR	Sensitivity: 0.92 Specificity: 0.7	NR	Cross‐validation	NR	NR
Karter et al. [22]	Univariate analysis followed by multivariate analysis	Number of prior episodes of hypoglycaemia‐related utilisation, insulin use, sulphonylurea use, prior year ED use, chronic kidney disease stage, age (6)	0.830	H‐L test *p* = 0.310	Random split verification	External validation of 2 independent samples	Hypoglycaemia Risk Stratification Tool
Chow et al. [23]	Stepwise selection	Glycemic management, age, race, education, waist circumference, medications (insulin, antihypertensive, HMG‐CoA reductase inhibitors, sulphonylurea, biguanide and meglitinide), years since diabetes diagnosis, history of hypoglycaemia in the last week, systolic blood pressure, diastolic blood pressure, serum creatinine, urinary albumin creatinine ratio (12)	0.782	Not reported	Cross‐validation, Bootstrap	NR	Map risk scores to predictors
Kyungdo Han et al. [24]	Univariate analysis followed by multivariate analysis	Older age (≥ 65 years), female sex, current smoker, drinking, low body mass index, lack of exercise, previous SH events, insulin or multiple oral hypoglycaemic agent use, presence of hypertension or chronic kidney disease, longer duration of diabetes, low or high glucose level, high Charlson Comorbidity Index score (14)	0.871	Calibration plot	Bootstrap	NR	Interactive web platform for automated computational forecasting
Misra‐Hebert et al. [25]	Backward stepwise selection	Age, gender, race, median income by zip code, Charlson comorbidity index, HbA1c, body mass index, diabetes medication classes, comorbidities, months since first non‐severe hypoglycaemia, history of non‐severe hypoglycaemia within 3 months (11)	0.890	Calibration plot	Cross‐validation, Bootstrap	NR	Web‐based hypoglycaemia risk prediction calculator based on scores
Heller et al. [26]	Demographic and treatment information	Insulin treatment, eGFR at baseline, previous stroke, diabetes duration, sex (male), LDL:HDL ratio at baseline, HbA1c, diastolic blood pressure, hepatic impairment, smoking status (10)	0.630	Calibration plot	Bootstrap	External validation	NR
Hu et al. [27]	Stepwise logistic regression after univariate analysis	Fasting insulin, fasting blood glucose, total treatment time (3)	0.666	H‐L test *p* = 0.3662	Bootstrap	NR	NR
T.Elhadd et al. [28]	NR	BMI, HbA1c, age, gender, SU use, Ramadan, hour of the day, day of week, part of the day, physical activity (10)	*R* ^2^: 0.548, MAE: 30.30	NR	5‐fold cross‐ validation	NR	NR
Crutzen et al. [29]	Multifactorial analysis	Male gender, age, total drug count, glucose‐lowering drug count, sulphonylurea use, insulin use, pre‐mixed insulin use, insulin count, insulin duration, antidepressant use (10)	0.71	NR	Cross‐validation	NR	NR
Kyi et al. [30]	Univariate analysis	Admission dysglycaemia, glycated haemoglobin ≥ 8.1%, glucose‐lowering treatment regimen containing sulphonylurea or insulin, glucocorticoid medication treatment, Charlson Comorbidity Index score, number of observed days (7)	0.806	NR	Calculation (Split Sample Method)	NR	Risk stratification tools
Zuo et al. [31]	Univariate analysis followed by multivariate analysis	BMI, length of stay, diabetes progression, peripheral vasculopathy, HbA1c, TG (6)	0.867	H‐L test *p* = 0.071	Random split verification	NR	NR
Han et al. [32]	Stepwise logistic regression after univariate analysis	Duration of diabetes ≥ 10 years, BMI < 18.5 kg/m^2^, SDBG ≥ 3.0 mmol/L, preoperative hypoglycaemic regimen of insulin subcutaneous (4)	0.715	H‐L test *p* = 0.765	Bootstrap	NR	Scoring system
Yang et al. [33]	Select and filter important variables using the feature importance parameter	Alanine aminotransferase, activated partial thromboplastin time, aspartate aminotransferase, C‐reactive protein, DBP, FIB, GLB, glucose, haemoglobin, HbA1c, HDL, heart rate, iod‐Nateglinide, LDL, pulse, PCT, blood platelet count, prothrombin time, respiratory rate, RBC, SBP, body temperature, total protein, prothrombin time, uric acid, insulin, sex, creatinine, urea, age, albumin, BMI, sodium, potassium, chloride, WBC, cholesterol (37)	0.822^a^	Calibration plot	Cross‐validation	External validation	NR
Liu et al. [34]	Univariate analysis followed by multivariate analysis	Operation time, BMI, pre‐operative C‐peptide, preoperative FBG (4)	0.745	H‐L test *p* = 0.606	NR	NR	Scoring system
Zhang et al. [35]	Literature review and expert meeting methodology	Number of hypoglycaemic episodes in 1 month, HDL, heart disease, diabetes education, combination medications, age, duration of diabetes, restricted staple food intake, HbA1c, sex (10)	0.823	Calibration plot	Bootstrap	30% sample size for external validation AUC = 0.859	NR
Zheng [36]	Meta‐analysis followed by univariate analysis	LR: postprandial 2 h C‐peptide, blood urea nitrogen, fasting C‐peptide, waist circumference, alanine aminotransferase, aspartate aminotransferase, urinary creatinine, haemoglobin (8)	0.82	NR	5‐fold cross‐ validation	NR	NR
		XGboost: fasting insulin, urine creatinine, glycated serum protein, blood urea nitrogen, postprandial 2 h C‐peptide, total bilirubin, fasting C‐peptide, HDL (8)	0.91				
		DT: fasting insulin, postprandial 2 h C‐peptide, fasting C‐peptide, TG, urinary creatinine, blood urea nitrogen, glycosylated serum protein, waist circumference, oral hypoglycaemic agents (9)	0.84				
		RF: postprandial 2 h C‐peptide, fasting C‐peptide, fasting insulin, blood urea nitrogen, oral hypoglycaemic agents, haemoglobin, TG, urinary creatinine, glycated serum protein (9)	0.89				
Xu et al. [37]	Stepwise logistic regression after univariate analysis	Model 3 month: age, central obesity, thiazolidinediones combined with insulin, intensive insulin therapy, frequency of hypoglycaemia in the past year, hypoglycaemia prevention education (6) Model 6 month: age, central obesity, occupation, HbA1c, thiazolidinediones combined with insulin, intensive insulin therapy, frequency of hypoglycaemia in the past year, hypoglycaemia prevention education, IAH (9)	0.723^a^	H‐L test *P* = 0.928^a^	NR	NR	NR
Shao et al. [38]	Forward, backward stepwise selection	NR	0.996^a^	NR	Cross‐validation	External validation using data from 427 European Americans in the US	NR
Chen et al. [39]	Univariate analysis	LGBM: TBR, M value, LBGI, minimum blood glucose, diabetes duration, maximum blood glucose, CV, insulin, BGRI, SD (10)	0.869^a^	H‐L test, *P*: not reported	10‐fold cross‐ validation	NR	NR
Guo et al. [40]	Univariate analysis +multivariate analysis by Lasso Regression	History of hypoglycaemia, therapy (sulphonamide, insulin and its combination with other drugs), MMSE score, TG (4)	0.801	*p* = 0.212	Bootstrap	NR	Nomogram
Agraz et al. [41]	Expert knowledge‐ based feature selection, Automatic feature selection^b^	HbA1c, general diabetes education, FPG, insulin (4)	Negative projections: 0.873, positive projection: 0.300, specificity: 0.993, accuracy: 0.868	NR	Cross‐validation, Bootstrap	NR	APP
Gaikwad et al. [42]	Common Warnings and Literature Review	NR	0.821^a^	NR	Cross‐validation	NR	Scoring system
Hou et al. [10]	Univariate analysis +multivariate analysis by Lasso Regression	Gender, DBP, family per capita monthly income, alcohol consumption, number of hypoglycaemia episodes in recent 1 year, identification of hypoglycaemic symptoms, hypertension, hyperlipidaemia, polypharmacy, random C‐peptide, HbA1c	0.830	Brier Score: 0.156	Bootstrap	NR	Nomogram
Thomsen et al. [43]	Automatic feature selection^b^	NR	0.941	NR	Random split verification	External validation on a separate T2D data set	NR

*Note:* Automatic feature selection methods: utilise the Boruta, MRMR and LASSO methods as automatic feature selection algorithms.

Abbreviations: AdaBoost, adaptive Boosting; AUC, area under the receiver operating characteristic curve; BGRI, blood glucose risk index; BMI, body mass index; CGM, continuous glucose monitoring; Charlson, Charlson comorbidity index; CV, coefficient of variation; DBP, diastolic blood pressure; eGFR, estimated glomerular filtration rate; FBG, fasting blood glucose; FIB, fibrinogen; FPG, fasting plasma glucose; GLB, globulin; HDL, high‐density lipoprotein; H–L, Hosmer–Lemeshow; IAH, impaired awareness of hypoglycaemia; iod‐Nateglinide, iodine urea and Nateglinide; LBGI, low blood glucose index; LGBM, light gradient Boosting machine; LDL, low‐density lipoprotein; LR, logistic regression; LSTM, long‐short term memory; MAE, mean absolute error; Mini – mental state examination; *M* value, a weighted average glucose value; MMSE, NR, not reported; PCT, procalcitonin; RBC, red blood cell count; RF, random forest; SBP, systolic blood pressure; SD, the standard deviation; SU, sulphonylurea; SVM, support vector machine; TBR, percentage of time below the target glucose range; TG, triglyceride; WBC, white blood cell count; XGboost, eXtreme gradient boosting.

^a^ The study established multiple models based on different model building methods, and the results of the models with the best discrimination and calibration are listed in the table.

^b^ Expert knowledge‐based feature selection methods: Selection of variables as candidate characteristics on the basis of clinical relevance and expert specialisation.

**FIGURE 3:**
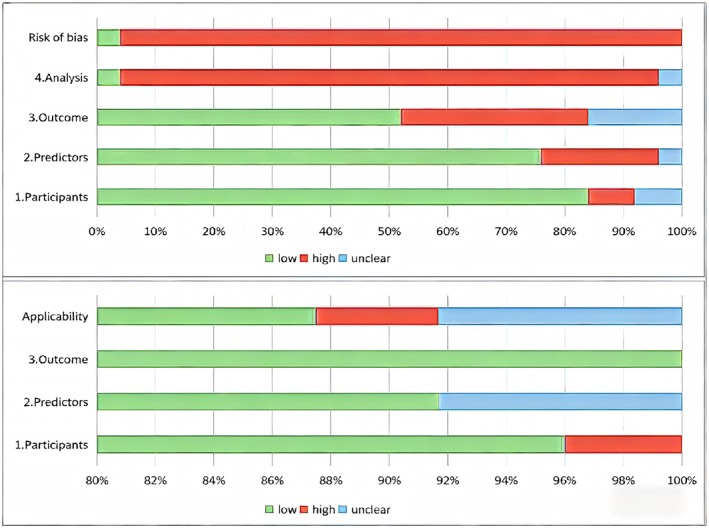
Summary results on risk of bias and applicability assessment (prediction model risk of bias assessment tool, PROBAST). Inclusion criteria: (1) The study reported the model's AUC along with its 95% confidence interval. (2) If a study developed models using multiple methods, only the model with the highest AUC was included. Exclusion criteria: (1) Studies with a cross‐sectional design. (2) Study categories with fewer than three eligible studies.

We used PROBAST to assess the ROB and applicability (Figure 3 and Table 4) of all 25 included studies. Twenty‐four studies (96%) were judged as high overall risk of bias. One study [24] had poor applicability and 2 studies [38, 43] had unclear applicability, with the remaining 22 studies showing good applicability.

**TABLE 4:** Risk of bias and clinical applicability of included studies (*n* = 25).

Study	ROB				Applicability			Overall	
	Participants	Predictors	Outcome	Analysis	Participants	Predictors	Outcome	ROB	Applicability
Murata et al. [20]	+	+	+	−	+	+	+	−	+
Sudharsan et al. [21]	+	−	+	−	+	+	+	−	+
Karter et al. [22]	−	+	−	−	+	+	+	−	+
Chow et al. [23]	+	+	−	?	+	+	+	−	+
Kyungdo Han et al. [24]	−	+	−	−	−	+	+	−	−
Misra‐Hebert et al. [25]	+	+	−	−	+	+	+	−	+
Heller et al. [26]	+	+	+	−	+	+	+	−	+
Hu et al. [27]	?	−	?	−	+	+	+	−	+
T. Elhadd et al. [28]	+	+	+	−	+	+	+	−	+
Crutzen et al. [29]	+	+	+	−	+	+	+	−	+
Kyi et al. [30]	+	+	+	−	+	+	+	−	+
Zuo et al. [31]	+	−	+	−	+	+	+	−	+
Han et al. [32]	+	−	+	−	+	+	+	−	+
Yang et al. [33]	+	+	+	−	+	+	+	−	+
Liu et al. [34]	+	+	?	−	+	+	+	−	+
Zhang et al. [35]	+	+	−	−	+	+	+	−	+
Zheng [36]	+	+	+	−	+	+	+	−	+
Xu et al. [37]	+	+	−	−	+	+	+	−	+
Shao et al. [38]	+	?	+	−	+	?	+	−	?
Chen et al. [39]	+	+	−	−	+	+	+	−	+
Guo et al. [40]	+	+	−	−	+	+	+	−	+
Agraz et al. [41]	+	+	+	−	+	+	+	−	+
Gaikwad et al. [42]	?	+	?	−	+	+	+	−	+
Hou et al. [10]	+	+	+	+	+	+	+	+	+
Thomsen et al. [43]	+	−	?	−	+	?	+	−	?

*Note:* + low ROB/low concern regarding applicability, − high ROB/high concern regarding applicability, ? unclear ROB/unclear concern regarding applicability.

Abbreviation: ROB: risk of bias.

### 3.1 Risk of Bias

In 24 studies with high risk of bias, the risks were mainly in areas of outcome and analysis domain. Among the 24 studies, in the outcome domain, 8 studies showed high risk of bias, and 4 studies had an unclear risk of bias, mainly because it was not possible to determine whether the predicted outcome was related to predictors. In the analysis domain, except for one study [23] which was not clear, the other 23 studies showed a high risk of bias, the main reasons being: (1) 13 studies had an insufficient number of sample sizes, only 2 studies reported the sample size calculation method, and the number of events per variable in the remaining studies was < 10 (EPV < 10) or the study did not report relevant information; (2) 13 studies did not report the handling of missing data and 4 studies directly deleted missing data; (3) 11 studies did not report calibration; (4) 4 studies selected predictors based on univariate analysis.

### 3.2 Risk of Applicability

22 studies showed good applicability, 1 study [24] reported poor applicability (participants limited aged ≥ 30 years) and 2 studies [38, 43] had unclear applicability (did not report predictor information).

**FIGURE 4:**
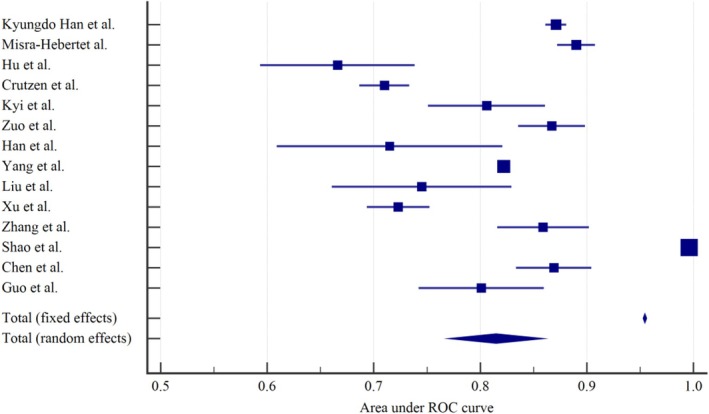
Forest plot of the random effects meta‐analysis of pooled AUC estimates for 16 studies.

Due to insufficient reporting on the development details of the models in the studies included, only the studies that reported the model's AUC and 95% confidence interval were included in the meta‐analysis. If there were studies that involved multiple methods for model development, we selected the model's best AUC for inclusion. Additionally, cross‐sectional studies predict prevalence rather than future risk and may overestimate model performance; therefore, they were also excluded from the meta‐analysis. Finally, 14 studies were eligible for synthesis. The pooled AUC was calculated using a random effects model, resulting in a value of 0.815 (95% CI: 0.765–0.861) (Figure 4). The *I* ^2^ value was 99.82% (*p* < 0.001, 95% CI: 99.80–99.84), indicating a high degree of heterogeneity among the studies. The results of publication bias assessment using Egger's test (*p* = 0.0948) suggest no significant publication bias in the present study.

**FIGURE 5:**
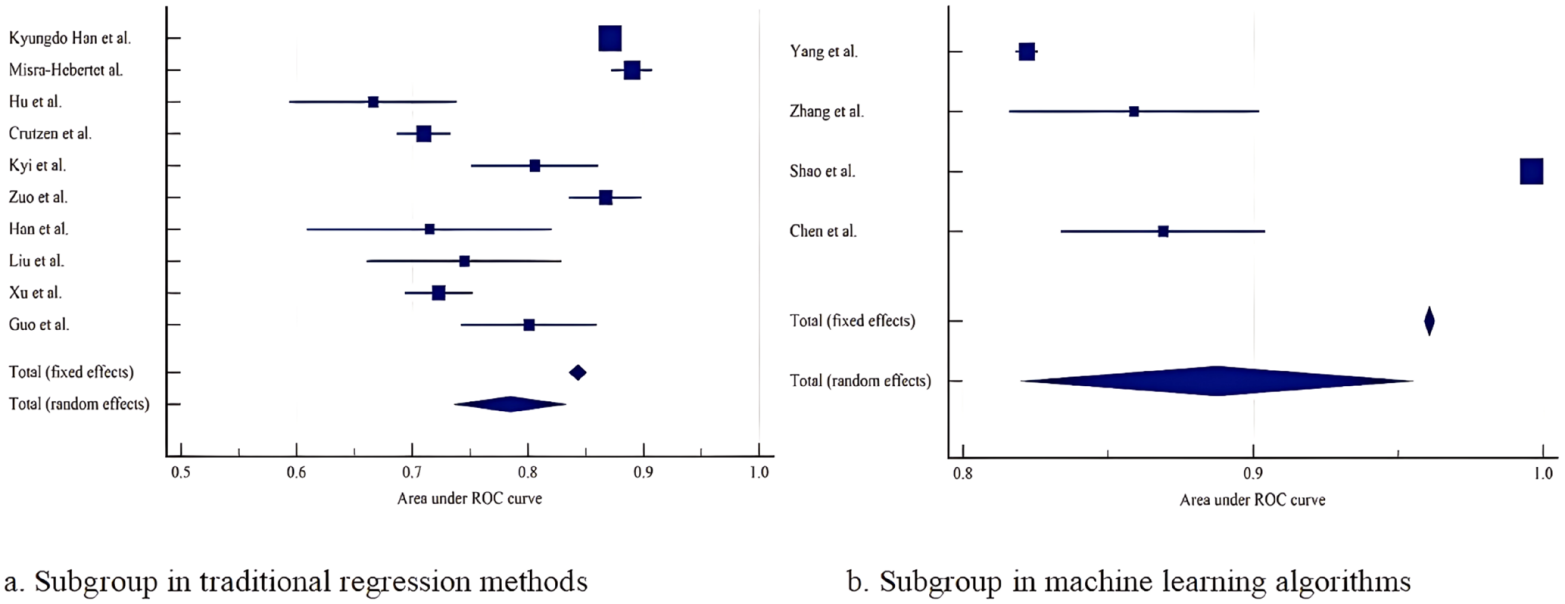
Forest plot of the random‐effects meta‐analysis for pooled AUC in subgroups of traditional regression methods and machine learning algorithms.

The high heterogeneity observed raises concerns regarding the validity of the pooled estimates. Considering that the underlying source of this substantial heterogeneity may be attributed to the differing methodologies employed for model construction, the 14 included studies were stratified into two subgroups based on their analytical approach: traditional regression methods (LR and COX) and machine learning algorithms (RF, XGBoost, LSTM, SVM, etc.). Separate meta‐analyses of the model AUC values were then conducted for each subgroup. Among the 14 included studies, 10 utilised traditional regression methods to develop models, while 4 employed machine learning algorithms. We conducted subgroup analyses based on this categorisation. The results showed that in the traditional regression subgroup, the pooled AUC calculated using a random‐effects model was 0.785 (95% CI: 0.737–0.832). High heterogeneity was observed among studies in this subgroup, with an *I* ^2^ value of 96.84% (*p* < 0.001, 95% CI: 95.55–97.76). In the machine learning algorithm subgroup, the pooled AUC calculated using a random‐effects model was 0.888 (95% CI: 0.821–0.954). High heterogeneity was also present in this subgroup, with an *I* ^2^ value of 99.95% (*p* < 0.001, 95% CI: 99.94–99.96; Figure 5).

## 4 Discussion

Research on hypoglycaemia risk prediction models for patients with T2DM began in 2004 [20]. Among 25 studies, 45 models included in this review, 11 studies (44%), 23 models (51.1%) were published in the past 2 years (2023–2024), which indicates that the field is in a rapid development stage. Nearly half of the models included were developed and validated based on the Chinese population, followed by Europe and the United States, suggesting that hypoglycaemia risk prediction models for patients with T2DM are gradually gaining global attention. Moreover, 11 of the 25 studies utilised large‐sample datasets and employed machine learning algorithms for model development. Large‐sample studies improve the robustness of model construction and validation, while machine learning offers distinct advantages in processing big data. These findings highlight the urgent need for an updated systematic review in this field.

Among the literature included in this study, 11 applied machine learning algorithms, 5 of them being from Chinese scholars. In recent years, China has issued a series of documents to promote the application of artificial intelligence and machine learning technology in the medical field. For example, ‘New Generation Artificial Intelligence Development Plan’ (2017) proposed to support the research and application of machine learning technology in clinical prediction models. ‘Guiding Principles for the Classification and Definition of Artificial Intelligence Medical Software Products’ (2021) proposed specific evaluation and approval criteria for clinical prediction model software based on machine learning. In addition, many Chinese universities have added ‘Big Data Management’ majors, and talents in machine learning have been cultivated. This may be the reason why the use of machine learning algorithms to build prediction models is increasing in recent years in China. The expansion of data sources and clinical demand drive are also reasons to promote the widespread application of machine learning in the medical field.

The prediction horizon of the model puts forward higher requirements for accuracy and reliability, which is important for the clinical value and applicability. Only 9 (36%) of the included studies reported the prediction horizon, and the prediction horizon ranged from 3 months to 1 year in 7 studies (77.8%). Considering hypoglycaemia risk prediction in patients with T2DM requires not only basic clinical data but also follow‐up data at different time points in the course of the disease, many of which change frequently (e.g., dietary intake, physical activity). An added consideration is the fact that since the occurrence of hypoglycaemia is an emergency event, the prediction horizon of the model should not be too long. We evaluated 35 models that demonstrated predictive performance in internal research or external validation, with reported AUC values ranging from 0.63 to 0.996. The pooled AUC value of the 16 models included in the meta‐analysis was 0.815 (95% CI: 0.765, 0.861). It can be considered that the prediction model has excellent combined discrimination for the risk of hypoglycaemia in T2DM. However, the heterogeneity among the models was high, which may be related to the differences in population, predictors and methodology of the different models.

‘Developing clinical prediction models: a step‐by‐step guide’ states that published prediction models often have methodological limitations and insufficient clinical practicability, and puts forward a 13‐step process for developing and validating clinical prediction models [14]. Our review followed this 13‐step process to systematically evaluate hypoglycaemia prediction models in patients with T2DM. The 25 studies included were all newly constructed models and 24 studies had a high risk of bias, and there were no studies that optimised existing models. Bias risk review is an important part of the systematic evaluation of risk prediction models [17]. The Guide suggests that if an existing model has a low risk of bias (according to PROBAST) and is applicable to the review question, in this case, assessing its validity for the intended setting may be more appropriate than developing a new model [14]. However, 44 hypoglycaemia risk prediction models showed high risk of bias, and the risk was mainly concentrated in the outcome and analysis domain, which was manifested in four shortcomings: whether the outcome was determined without knowledge of predictor information, insufficient sample size, missing data were not handled appropriately and inappropriate screening methods of predictors were used.

When collecting hypoglycaemia data, clinical researchers hardly avoid exposure to predictor data, and as such data are primarily sourced from electronic medical records or face‐to‐face collection, this may lead to bias in outcome assessment. In the future, blinding procedures should be incorporated into data extraction and model development to ensure the scientific validity and accuracy of the results. Regarding the high risk of bias due to inadequate sample size, 12 of the included studies (48%) had fewer than 1000 cases and only 3 out of the 25 studies [10, 35, 39] reported their sample size calculation methods. Some models were developed with few samples relative to the number of predictors, leading to overfitting—where a model performs well on the original dataset but poorly on new data. Therefore, developing prediction models on adequately sized samples is critical for achieving stable model performance. Minimum sample size calculations should consider multiple dimensions, including discrimination, calibration and overall net benefit. Estimating sample size before model development can help prevent imprecision and overfitting from the outset [44]. Concerning bias arising from inappropriate handling of missing data, 13 models (28.9%) simply deleted records with missing values, which may discard valuable information and affect both model development and evaluation. Multiple imputation is a widely recommended approach during model building, as it properly accounts for the uncertainty associated with missing data [45]. On the bias due to suboptimal predictor selection, 4 studies selected variables based solely on univariate analysis, without considering inter‐predictor relationships, potentially omitting important predictors. It is advisable to use regularisation methods—such as Ridge Regression, LASSO or Elastic Net—to control model complexity and avoid overfitting [46]. Additionally, incorporating well‐established predictors and clinically plausible variables into the selection process is essential for developing stable and generalisable models. Notably, the studies included in this research exhibited significant variations in the definition of hypoglycaemia, primarily reflected in four aspects: blood glucose thresholds, inclusion of symptoms, criteria for severity and data sources. This lack of standardised definition contributed to the high heterogeneity observed in the meta‐analysis and represents a limitation of this study. Future research should focus on establishing and adopting a unified definition and reporting standard for hypoglycaemia, which would improve study homogeneity and results comparability in this field.

The 45 hypoglycaemia risk prediction models included in this study contain multiple independent predictors, and the predictors vary widely among studies. Moreover, the models constructed using a machine learning algorithm did not report OR value of the predictors, so further meta‐analysis of predictors is not possible. Therefore, we tallied the frequency of each predictor across the models. The top five predictors were: insulin use (16 times), sulphonylurea use (12 times), age (10 times), glycated haemoglobin (HbA1c, 10 times) and history of hypoglycaemia (9 times). Insulin lowers blood glucose by inhibiting hepatic glucose output and promoting peripheral glucose utilisation. When its dosage is excessive relative to food intake and energy expenditure, it predisposes to hypoglycaemia [47]. Sulphonylureas stimulate insulin secretion; particularly in the fasting state, they may cause inappropriate insulin release, leading to hypoglycaemia [48]. In elderly patients, the risk of hypoglycaemia is increased due to diminished counterregulatory hormone responses, reduced or atypical awareness of hypoglycaemic symptoms, which often leads to delayed recognition and management. A low HbA1c level or its rapid decline over a short period often indicates excessive treatment intensity or unstable glycaemic control, thereby raising the risk of hypoglycaemia [47]. A history of hypoglycaemia can lead to hypoglycaemia‐associated autonomic failure, impairing the body's defence mechanisms against subsequent hypoglycaemic episodes and increasing the likelihood of recurrent events [49].

In terms of model validation, 43 models (95.6%) included were internally validated, the vast majority of which were cross‐validated using the bootstrapping method, and 5 models [22, 30, 31, 35, 43] used the random split method, which may lead to high risk of overfitting. Efron et al. [50] suggested using *k*‐fold and bootstrapping methods for cross‐validation, which help to reduce the randomness of data partitioning and more comprehensively assess the generalisation ability of the model, but this requires a larger sample size. The lack of external validation impedes the repeatability and clinical generalisation ability of the model. Among the 25 included studies, only 6 studies conducted external validation, of which 4 reported on the methods and processes of external validation. External validation of models should be performed on new datasets, and it is best to have independent researchers who were not involved in the development of the original model provide guidance on external validation methods [14]. These 4 externally validated studies applied new datasets, but did not provide information on the researchers' situation. Overall, we believe that although multiple risk prediction models for hypoglycaemia in T2DM patients have been developed, there is still a lack of a recognised and authoritative prediction model that can be recommended as a guide.

Subgroup analysis preliminarily suggests that, among the currently included studies, models developed using machine learning algorithms performed better on average in distinguishing hypoglycaemia than those built with traditional regression methods. However, both subgroups exhibited extremely high heterogeneity, with the machine learning subgroup showing particularly pronounced heterogeneity. This indicates a lack of standardisation in research methodologies in this field, making direct comparison and meta‐analysis of results difficult. The heterogeneity may originate from the substantial diversity inherent in machine learning approaches, such as differences in algorithm types, hyperparameter configurations and feature selection methods. Additionally, the inherent opacity and limited interpretability of machine learning algorithms may also contribute to the high degree of heterogeneity.

In recent years, with the development of artificial intelligence, machine learning algorithms, as a branch of artificial intelligence, have developed rapidly and been widely used in the field of clinical prediction. Among the 25 studies included, 11 studies constructed models using machine learning algorithms and the models all showed good performance. However, studies have shown that machine learning algorithms may not have additional performance advantages compared to traditional methods [51]. In addition, machine learning algorithmic models may also suffer from poor data cleaning, unsatisfactory tuning parameters, low model adaptability and difficulties in computerisation for clinical staff. Compared with traditional regression models, the construction process and validation of machine learning algorithm models are often not transparent enough and are difficult to explain clearly. When constructing a hypoglycaemia‐related prediction model, the practical applicability of the model should be considered and multiple algorithms should be tried when building the model, as much as possible. Then, various factors (e.g., variable selection, data collection, data processing) affecting the implementation of the model should be considered, then combined with medical expertise, clinical practice, previous experience and statistical judgement, to select the best model [52].

In addition, we recommend focusing on the interpretive aspects of the model when using machine learning algorithms to construct clinical prediction models. SHAP is currently the most commonly used interpretive framework, which quantifies the contribution of each feature in the model to the final prediction of the observed results, using a combination of prediction models based on all possible feature subsets [53], which would greatly aid in the interpretability of the model.

There are several limitations in the review. Forty‐four included models (97.8%) were rated as having high risk of bias, which reduces the credibility of our systematic review. This also implies that there is an urgent need for high‐quality and recognised prediction models of T2DM hypoglycaemia. It is recommended that future studies strictly follow the methodological guidelines for constructing prediction models. Besides, since risk of bias tools for machine learning models have not been published, we used PROBAST to assess the risk of bias for all included studies, which may not be appropriate for evaluating machine learning models.

## 5 Conclusions

The systematic review evaluated the quality and performance of 45 existing T2DM hypoglycaemia prediction models from 25 studies. The results showed that despite the good predictive performance of the current models, most of them have a high risk of bias. Their reported performance may appear optimistic in a specific dataset, but most of them lack external validation and the generalisation ability of the models is uncertain. We believe that these models are currently not suitable for clinical practice recommendations. In the future, the development and validation of predictive models should strictly follow rigorous methodological guidelines and reporting guidelines, and existing models can also be optimised and externally validated in different regions and populations to evaluate their feasibility and applicability.

## Author Contributions

Y.W. authored the first draft of the review protocol, contributed to the development of the search strategy, undertook the screening and selection of articles, extracted data, synthesised results and authored the first draft of the manuscript. Y.W., Y.L., X.B. and J.B. provided expertise in developing and performing the searches and approved the final manuscript for submission. Y.L. had the idea for the review, secured funding, edited and approved the review protocol, contributed to the development of the search strategy, synthesised results, edited and approved the final manuscript for submission. Y.W., J.B. and X.B. accessed and verified the data. Y.W., Y.L. and J.B. made the decision to submit the manuscript for publication.

## Funding

The authors have nothing to report.

## Ethics Statement

The authors have nothing to report.

## Conflicts of Interest

The authors declare no conflicts of interest.

## Supporting information


**Data S1:** This file presents the PROSPERO registration protocol (CRD420251031980) and the detailed search strategies for nine electronic databases (PubMed, Cochrane Library, CINAHL, Web of Science, ProQuest, Sinomed, CNKI, Wanfang and VIP).

## Data Availability

The data that supports the findings of this study are available in the Supporting Information of this article.
