# Supplementary material for: Hypoglycaemia Risk Prediction Models for Type 2 Diabetes: A Systematic Review and Meta‐Analysis
Source: Endocrinol Diabetes Metab. 2026 Apr 29;9(3):e70227. doi: 10.1002/edm2.70227 (PMC13125951; doi:10.1002/edm2.70227)
Supplement: Supplementary file 1 — Data S1: This file presents the PROSPERO registration protocol (CRD420251031980) and the detailed search strategies for nine electronic databases (PubMed, Cochrane Library, CINAHL, Web of Science, ProQuest, Sinomed, CNKI, Wanfang and VIP). [file EDM2-9-e70227-s001.pdf]

NIHR | National Institute for Health and Care Research

PROSPERO
International prospective register of systematic reviews

# Hypoglycemia risk prediction models for type 2 diabetes: A systematic review and meta-analysis

*yiwen wei, yu liu*

**Citation**
yiwen wei, yu liu. Hypoglycemia risk prediction models for type 2 diabetes: A systematic review and meta-analysis. PROSPERO 2025 CRD420251031980. Available from https://www.crd.york.ac.uk/PROSPERO/view/CRD420251031980.

## REVIEW TITLE AND BASIC DETAILS

### Review title

Hypoglycemia risk prediction models for type 2 diabetes: A systematic review and meta-analysis

### Condition or domain being studied

*Hypoglycaemia Due To Type 2 Diabetes Mellitus*

### Rationale for the review

The number of hypoglycemia risk prediction models for patients with type 2 diabetes mellitus (T2DM) is increasing. We systematically searched the existing research on hypoglycemia risk prediction models in patients with T2DM and standardized the evaluation of their risk bias and applicability based on the guide for prediction model development and summarized and critically evaluated their features and predictive performance.

### Review objectives

The review provides a comprehensive and up to date review on hypoglycemia risk prediction model in patients with T2DM.The review found the development and validation of the existing hypoglycemia risk prediction models for T2DM have not been standardized, and there are significant methodological defects and high bias risks. It is necessary to optimize existing hypoglycemic prediction models or develop new models based on strict methodological guidance, and external validation should be carried out.

### Keywords

Type 2 diabetes mellitus; Hypoglycemia; Risk prediction model; Systematic review

### Country

China

## ELIGIBILITY CRITERIA

### Population

*Included*
Patients with diagnosed type 2 diabetes

*Excluded*
Type 1 diabetes mellitus, gestational diabetes mellitus, or other special diabetes mellitus.

### Intervention(s) or exposure(s)

*Included*
All available hypoglycemia risk prediction models.

### Comparator(s) or control(s)

This review does not have any comparators

### Study design

Only randomized study types will be included.

*Included*
cohort studies, cross-sectional studies, and randomized controlled studies.

*Excluded*
Reviews, commentaries, conference abstracts, and news reports.

### Context

The contexts in which risk prediction models are used, including inpatient, outpatient, acute care, community-based, and home-based settings.

## TIMELINE OF THE REVIEW

### Date of first submission to PROSPERO

15 April 2025

### Review timeline

Start date: 13 April 2025. End date: 1 June 2025.

### Date of registration in PROSPERO

15 April 2025

## AVAILABILITY OF FULL PROTOCOL

### Availability of full protocol

A full protocol has not been written.

## SEARCHING AND SCREENING

### Search for unpublished studies

Only published studies will be sought.

### Main bibliographic databases that will be searched

The main databases to be searched are *CINAHL - Cumulative Index to Nursing and Allied Health Literature*, *CLIB - The Cochrane Library*, *Embase - Embase via Ovid*, *Embase.com* and *PubMed*.

### Search language restrictions

The review will only include studies published in English.

### Search date restrictions

Databases will be searched for articles published before 1 June 2025, there are no restrictions on search start date.

### Other methods of identifying studies

Other studies will be identified by: *contacting authors or experts* and *searching dissertation and thesis databases*.

### Link to search strategy

A full search strategy has been uploaded to PROSPERO. The PDF may be accessed through this link https://www.crd.york.ac.uk/PROSPEROFILES/4b36c57e77ae4c3234fd01a1d65a3ba3.pdf.

### Selection process

Studies will be screened independently by at least two people (or person/machine combination) with a process to resolve differences.

### Other relevant information about searching and screening

None

## DATA COLLECTION PROCESS

### Data extraction from published articles and reports

Data will be extracted independently by at least two people (or person/machine combination) with a process to resolve differences.

Authors will be asked to provide any required data not available in published reports.

### Study risk of bias or quality assessment

Risk of bias will be assessed using: *PROBAST*

Data will be assessed independently by at least two people (or person/machine combination) with a process to resolve differences.

Additional information will be sought from study investigators if required information is unclear or unavailable in the study publications/reports.

### Reporting bias assessment

Risk of bias due to missing results will be assessed

### Certainty assessment

The data extraction process was conducted by two reviewers. In cases of disagreement during data extraction, a third reviewer was invited to reach a consensus through discussion.

## OUTCOMES TO BE ANALYSED

### Main outcomes

The occurrence of hypoglycemia, including symptomatic hypoglycemia, asymptomatic hypoglycemia, and hypoglycemia-related events

### Additional outcomes

There are no additional outcomes.

## PLANNED DATA SYNTHESIS

### Strategy for data synthesis

A meta-analysis of the area under the curve (AUC) values from models was conducted using MedCalc software (version20.0). Heterogeneity was tested using the I2 index and Cochrane Q test. Fixed or random effects models were used based on the heterogeneity of the analysis results, and Egger's test was used to identify publication bias, with p > 0.05 indicating a low likelihood of publication bias

## CURRENT REVIEW STAGE

### Stage of the review at this submission

| Review stage | Started | Completed |
|---|---|---|
| Pilot work | ✓ | |
| Formal searching/study identification | ✓ | |
| Screening search results against inclusion criteria | | |
| Data extraction or receipt of IPD | | |
| Risk of bias/quality assessment | | |
| Data synthesis | | |

### Review status

The review is currently planned or ongoing.

### Publication of review results

Results of the review will be published in English.

## REVIEW AFFILIATION, FUNDING AND PEER REVIEW

### Review team members

**Mr yiwen wei** (review guarantor and contact) Beijing University of Chinese Medicine. China.

No conflict of interest declared.

**Mrs yu liu**. Beijing University of Chinese Medicine. China.

No conflict of interest declared.

### Named contact

**Mr yiwen wei** (1047686527@qq.com). Beijing University of Chinese Medicine. China.

### Review affiliation
Beijing University of Chinese Medicine,

### Funding source
Review has no specific/external funding but is supported by guarantor/review team (non-commercial) institutions.

### Peer review
There has been no peer review of this planned review.

## ADDITIONAL INFORMATION

### Review conflict of interest
Declared individual interests are recorded under team member details.. No additional interests are recorded for this review.

### Medical Subject Headings
Diabetes Mellitus, Type 2; Hypoglycemia; Hypoglycemic Agents; Risk Factors

## SIMILAR REVIEWS

### Check for similar records already in PROSPERO
*PROSPERO identified a number of existing PROSPERO records that were similar to this one (last check made on 13 April 2025). These are shown below along with the reasons given by that the review team for the reviews being different and/or proceeding.*

- Hypoglycemia risk prediction model for diabetes mellitus: a systematic review and critical appraisal [published 13 September 2021] [CRD42021272941]. The review was judged **not to be similar**
- Prediction models for hypoglycemia in people with type 2 diabetes: systematic review and critical appraisal [published 6 January 2022] [CRD42022296699]. The review was acknoweldged as **similar** but the authors opted to continue because *the review looks at additional or different outcomes, the review will be more up to date, the review uses improved methods*
- Risk prediction models for hypoglycemia in diabetes patients: a systematic review [published 1 February 2021] [CRD42021232959]. The review was judged **not to be similar**

### PROSPERO version history

- Version 1.0, published 15 Apr 2025

### Disclaimer
The content of this record displays the information provided by the review team. PROSPERO does not peer review registration records or endorse their content.

PROSPERO accepts and posts the information provided in good faith; responsibility for record content rests with the review team. The guarantor for this record has affirmed that the information provided is truthful and that they understand that deliberate provision of inaccurate information may be construed as scientific misconduct.

PROSPERO does not accept any liability for the content provided in this record or for its use. Readers use the information provided in this record at their own risk.

Any enquiries about the record should be referred to the named review contact

| Database | Search strategies |
| --- | --- |
| **PubMed** | ((MH "Diabetes Mellitus, Type 2") OR "type 2 diabetes" OR "diabetes, type 2" OR "T2DM" OR "2-dm") AND ((MH "Hypoglycemia") OR "hypoglycemia" OR "glycopenia") AND ("risk prediction" OR "risk evaluation" OR "risk assessment" OR "prediction model" OR "predict" OR "tool" OR "Scale" OR "score") |
| **Cochrane Library** | ("type 2 diabetes" OR "diabetes, type 2" OR "T2DM" OR "2-dm") AND ("hypoglycemia" OR "glycopenia") AND ("risk prediction" OR "risk evaluation" OR "risk assessment" OR "prediction model" OR "predict" OR "tool" OR "Scale" OR "score") |
| **CINAHL** | ((MH "Diabetes Mellitus, Type 2") OR SU "type 2 diabetes" OR SU "diabetes, type 2" OR SU "T2DM" OR SU "2-dm") AND ((MH "Hypoglycemia") OR (SH "hypoglycemia") OR SU "glycopenia") AND ( SU "risk prediction" OR SU "risk evaluation" OR SU "risk assessment" OR SU "prediction model" OR SU "predict" OR SU "tool" OR SU "Scale" OR SU "score") |
| **Web of Science** | AB=("type 2 diabetes" OR "diabetes, type 2" OR "T2DM" OR "2-dm") AND AB=( "hypoglycemia" OR "glycopenia") AND AB=("risk prediction" OR "risk evaluation" OR "risk assessment" OR "prediction model" OR "predict" OR "tool" OR "Scale" OR "score") |
| **ProQuest** | AB=("type 2 diabetes" OR "diabetes, type 2" OR "T2DM" OR "2-dm") AND AB=( "hypoglycemia" OR "glycopenia") AND AB=("risk prediction" OR "risk evaluation" OR "risk assessment" OR "prediction model" OR "predict" OR "tool" OR "Scale" OR "score") |
| **Sinomed** | ("2 型糖尿病"[摘要] OR "糖尿病 2 型"[摘要]) AND ("低血糖"[摘要]) AND (" 风险评估"[摘要] OR "风险预测"[摘要] OR "风险因素"[摘要] OR "预测模型 "[摘要] OR "预警模型"[摘要] OR "预测"[摘要] OR "模型"[摘要] OR "工具 "[摘要] OR "量表"[摘要]) |
| **中国知网** | TKA="2 型糖尿病" + "糖尿病 2 型" AND TKA="低血糖"AND TKA="风 险评估" + "风险预测" + "风险因素" + "预测模型" + "预警模型" + "预测" + " 模型" + "工具" + "量表” |
| **万方** | 摘要:("2 型糖尿病" OR "糖尿病 2 型") AND 摘要:("低血糖" ) AND 摘要:("风 险评估" OR "风险预测" OR "风险因素" OR "预测模型" OR "预警模型" OR " 预测" OR "模型" OR "工具" OR "量表" ) |
| **维普** | (R="2 型糖尿病" OR R="糖尿病 2 型") AND (R="低血糖") AND (R="风险评估 " OR R="风险预测" OR R="风险因素" OR R="预测模型" OR R="预警模型" OR R="预测" OR R="模型" OR R="工具" OR R= "量表") |
